# Food Products and Their Chief Adulterants

**Published:** 1891-06

**Authors:** 


### Food Products and Their Chief Adulterants.

| Food Product. | Adulterant. |
|---|---|
| Milk | Water, removal of cream, addition of oleo oil or lard to skimmed milk. |
| Butter | Water, salt, foreign fats, artificial coloring matter. |
| Cheese | Lard, oleo-oil, cotton-seed oil. |
| Olive Oil | Cotton-seed and other vegetable oils. |
| Beer | Artificial glucose, malt and hop substitutes, sodium bicarbonate, salt, antiseptics. |
| Syrup | Artificial glucose. |
| Honey | Artificial glucose, cane sugar. |
| Confectionery | Artificial glucose, starch, artificial essences, poisonous pigments, terra alba, gypsom |
| Wines, liquors | Water, spirits, artificial coloring matter, burnt sugar, etc. |
| Vinegar | Water, other mineral or organic acid. |
| Flour, bread | Other meals, alum. |
| Bakers' chemicals | Starch, alum. |
| Spices | Flour, starches of various kinds, tumeric. |
| Coca and Chocolate | Sugar, starch, flour. |
| Coffee | Chicory, peas, beans, rye, corn, wheat, coloring matter. |
| Tea | Exhausted tea-leaves, foreign leaves, tannin, indigo, Prussian blue, tumeric, gypsom, soap-stone, sand. |
| Canned goods | Metallic poisons. |
| Pickles | Salts of copper. |

### Cures for Neuralgia of the Head.

An English physician has recently asserted that headaches and neuralgia could be stopped by blowing a solution of salt up the nose. Whew!

